# Supplementary material for: Content-rich biological network constructed by mining PubMed abstracts
Source: BMC Bioinformatics. 2004 Oct 8;5:147. doi: 10.1186/1471-2105-5-147 (PMC528731; doi:10.1186/1471-2105-5-147)
Supplement: Additional File 2 — The original results of the above study (non-essential files are deleted to keep the file size under the limit set by BMC bioinformatics). [file 1471-2105-5-147-S2.bz2 › chilibotAdditionalFile2/dip30/671ID11110785E11073/html/CCR3_CXCL10.html]

 


 **CCR3** and **CXCL10** 
  
Found 37 abstracts in PubMed, retrieved 30.  
 

 What does Google say? 
 PDF only 
| .edu only 

---

**Interactive relationship** (e.g. stimulation, inhibition, etc)

**Neutral relationship**- The ligands of CXC chemokine receptor 3  [ **CCR3** ] , I TAC, Mig, and IP10  [ **CXCL10** ] , are natural antagonists for  **CCR3** .  Ref: 11110785 J Biol Chem, 2001
- IP10  [ **CXCL10** ] , MIG and TARC positive HD cases contained higher numbers of Th2 lymphocytes ST2  **CCR3**  or CCR4 positive lymphocytes, compared to IP10  [ **CXCL10** ] , MIG and TARC negative HD cases p <.  Ref: 11920617 Int J Cancer, 2002
- I TAC, Mig, and IP10  [ **CXCL10** ]  compete for the binding of eotaxin to  **CCR3**  bearing cells and inhibit migration and Ca 2 changes induced in such cells by stimulation with eotaxin, eotaxin 2, MCP 2 monocyte chemottractant protein 2, MCP 3, MCP 4, and RANTES regulated on activation normal T cell expressed and secreted.  Ref: 11110785 J Biol Chem, 2001
- **CCR3**  functional responses are regulated by both CXCR3 and its ligands CXCL9,  **CXCL10**  and CXCL11.  Ref: 12884299 Eur J Immunol, 2003
- An unusual structural feature of IP 10  [ **CXCL10** ]  was identified that may provide the basis for the ability of IP 10  [ **CXCL10** ]  to bind both CXCR3 and  **CCR3** .  Ref: 12173928 Biochemistry, 2002
- Immunohistochemical staining was performed using antibodies for CXCR3 a specific receptor for IP10  [ **CXCL10** ]  and MIG, which is characteristic of Th1 helper phenotype and  **CCR3** , CCR4 and ST2, which are features of Th2 cells.  Ref: 11920617 Int J Cancer, 2002
- IP 10  [ **CXCL10** ]  and Mig share a common functional receptor on activated T lymphocytes, named CXC chemokine receptor 3  [ **CCR3** ]  CXCR3.  Ref: 11134180 J Clin Invest, 2001
- CXC chemokine receptor 3  [ **CCR3** ]  CXCR3, predominately expressed on memory activated T lymphocytes, is a receptor for both IFN gamma inducible protein 10 gamma IP 10  [ **CXCL10** ]  and monokine induced by IFN gamma Mig.  Ref: 10903763 J Immunol, 2000
- Type 1 chemokines CXC chemokine ligand CXCL 9 and  **CXCL10**  monokine induced by IFN gamma and IFN gamma inducible protein of 10 kDa CXCR3 ligands, and type 2 chemokines CC chemokine ligand CCL 11 eotaxin  **CCR3**  ligand, CCL17 thymus and activation regulated chemokine CCR4 ligand and CCL22 monocyte derived chemokine CCR4 ligand were readily detectable in the plasma of most participants.  Ref: 12407016 Int Immunol, 2002
- CXC chemokine receptor 3  [ **CCR3** ]  CXCR3, which is known to be expressed predominately on memory and activated T lymphocytes, is a receptor for both interferon gamma IFN gamma inducible protein 10 gamma IP 10  [ **CXCL10** ]  and monokine induced by IFN gamma Mig.  Ref: 10942362 Blood, 2000
- CXC chemokine receptor 3  [ **CCR3** ]  expression on CD34 hematopoietic progenitors from human cord blood induced by granulocyte macrophage colony stimulating factor chemotaxis and adhesion induced by its ligands, interferon gamma inducible protein 10  [ **CXCL10** ]  and monokine induced by interferon gamma.  Ref: 10942362 Blood, 2000

**Non-interactive relationship** (e.g. studied together, co-existance, homology, etc.)

- The upregulation of the CXCR3 ligand IP 10  [ **CXCL10** ] , as well as CCR5 and its ligands, in the absence of  **CCR3**  and CCR8 is indicative that renal allograft rejection is primarily the result of a Th1 type immune response.  Ref: 11228176 Am J Kidney Dis, 2001
- O 3 increased lung protein levels of  **CXCL10** , CCL7, and  **CCR3**  CCL7R.  Ref: 11777981 J Immunol, 2002
- Both thymus and activation regulated chemokine TARC CCL17, a ligand for CC chemokine receptor 4 that is mainly expressed on Th2 type cells, and interferon induced protein of 10kDa IP 10  [ **CXCL10** ]   **CXCL10** , a ligand for CXC chemokine receptor 3  [ **CCR3** ]  that is mainly expressed on Th1 type cells, are produced by keratinocytes after the stimulation with the primary cytokines such as tumor necrotic factor alpha TNF alpha and or interferon gamma IFN gamma.  Ref: 12441140 Cytokine, 2002
- Consistent with these results, infected cells from WT mice preferentially migrated in response to IP 10  [ **CXCL10** ]  CXCR3 ligand, while those from GKO mice migrated in response to eotaxin CCL11  **CCR3**  ligand.  Ref: 12875978 Am J Pathol, 2003
- Recently, certain chemokines and chemokine receptors have been preferentially associated with the selective recruitment in vitro of type 1 T cells, such as IP 10  [ **CXCL10** ]  and its receptor CXCR3, or type 2 T cells such as monocyte derived chemokine MDC and eotaxin and their receptors CCR4 and  **CCR3** .  Ref: 11238053 Am J Pathol, 2001
- The presence of chemokine receptor CCR5, CXCR3,  **CCR3**  positive cells and chemokine MCP1, MIP 1alpha, MIP 1beta, IP 10  [ **CXCL10** ]  positive cells in human periapical granulomas.  Ref: 11683586 Cytokine, 2001
- Using self organizing maps, we identified nine genes CD4,  **CCR3** , CCR5, LT beta, MIP 1 alpha, MIP 2, CD8 alpha, IP 10  [ **CXCL10** ] , and RANTES, each with a unique profile of transcriptional expression, that reproduce the differentiation of states of rejection in dendrograms.  Ref: 11818457 J Leukoc Biol, 2002
